# Smart Sensor-Based Monitoring Technology for Machinery Fault Detection

**DOI:** 10.3390/s24082470

**Published:** 2024-04-12

**Authors:** Ming Zhang, Xing Xing, Wilson Wang

**Affiliations:** 1 Automotive Engineering Department, Weifang Institute of Engineering, Qingzhou 262501, China; ming-zhang@emechsys.com; 2 Department of Mechanical and Mechatronics Engineering, Lakehead University, Thunder Bay, ON P7B 5E1, Canada; xxing2@lakeheadu.ca

**Keywords:** smart sensors, data acquisition, bearing fault detection, vibration signal analysis, variational mode decomposition

## Abstract

Rotary machines commonly use rolling element bearings to support rotation of the shafts. Most machine performance imperfections are related to bearing defects. Thus, reliable bearing condition monitoring systems are critically needed in industries to provide early warning of bearing fault so as to prevent machine performance degradation and reduce maintenance costs. The objective of this paper is to develop a smart monitoring system for real-time bearing fault detection and diagnostics. Firstly, a smart sensor-based data acquisition (DAQ) system is developed for wireless vibration signal collection. Secondly, a modified variational mode decomposition (MVMD) technique is proposed for nonstationary signal analysis and bearing fault detection. The proposed MVMD technique has several processing steps: (1) the signal is decomposed into a series of intrinsic mode functions (IMFs); (2) a correlation kurtosis method is suggested to choose the most representative IMFs and construct the analytical signal; (3) envelope spectrum analysis is performed to identify the representative features and to predict bearing fault. The effectiveness of the developed smart sensor DAQ system and the proposed MVMD technique is examined by systematic experimental tests.

## 1. Introduction

Rotating machines are commonly used in various industries, such as electric vehicles, aircraft, and machine tools. Machine component defects will influence machine performance quality and reliability. Based on this investigation, up to 75% of imperfections in small- and medium-size rotating machines are related to defects in rolling element bearings [1,2]. This work will focus on condition monitoring and fault diagnosis of rolling element bearings. Bearing defects can not only generate extra vibrations and noise, but also result in the degradation of machine operation accuracy or even the early breakdown of rotating machines. Thus, reliable machine condition monitoring systems are critically needed in industries to detect the bearing fault in its early stage so as to prevent machine performance degradation and improve operation efficiency and reliability.

A real-time machine condition monitoring system includes modules, such as data acquisition (DAQ) to collect signals, and signal processing for fault detection and diagnosis. Vibration signals will be used for bearing fault detection in this work due to its high signal-to-noise ratio. A traditional DAQ system uses a DAQ board to collect signals, which is not convenient in real industrial applications due to reasons such as the use of pre-conditioning hardware, sensor cable installation limitations, and high expenses. An alternative is to use smart sensor-based DAQ systems. A smart sensor integrates a sensing unit, pre-processing functionality, and wireless communication in one chip, controlled by a microcontroller unit (MCU) [3]. Although there are some smart vibration sensors available on the market [3,4,5], they cannot meet the comprehensive requirements of measurement range, frequency bandwidth and sensitivity, used in this monitoring application. Subsequently, the first objective of this work is to develop a new smart sensor-based DAQ system to collect vibration signals wirelessly.

Reliable fault detection in rolling element bearings remains a very challenging task in the research field of machine health monitoring and fault diagnosis. A bearing is not a component like a gear or shaft, but it is a system comprising components, such as an outer ring, an inner ring, a cage, and a set of rolling elements. Bearing defect-related feature properties depend on bearing geometry, defect location, and operating conditions. In addition, bearing features are also modulated by other strong vibration signals generated from vibratory sources such as gear meshing [1,6]. 

There are many signal processing techniques proposed in the literature for bearing fault detection [7,8,9,10]. The most commonly used approach is based on Fourier transform (FT) spectral analysis by examining health-related characteristic frequency components on the spectrums. However, FT analysis cannot be applied to analyze nonstationary signals whose spectral and statistical properties change with time [11,12]. Nonstationary and transient signals can be processed by analyzing multiresolution time–frequency information using techniques such as the Hilbert–Huang transform (HHT) [13]. The HHT uses empirical mode decomposition (EMD) to extract intrinsic mode functions and residual signature for signal property analysis; however, its signal decomposition approximation is sensitive to noise, which can affect the overall processing accuracy [14,15].

The variational mode decomposition (VMD) method decomposes the vibration signal into several signatures with different center frequencies by using a set of adaptive Wiener filters [16]. Although VMD has been used in the extraction of nonlinear features and machine fault detection [17,18], it still has some limitations. For example, it requires pre-choosing the number of modes and the bandwidth control parameters. Most of the available research in this area has focused on adaptability improvement and parameter optimization. For example, a prediction method was proposed in [19] for decomposition prediction by recursively modifying the related system parameters; however, it was an empirical algorithm and was difficult to implement in bearing fault detection, as different bearings have different system parameters and installation dynamics. A spectrum distribution-based VMD was presented in [20] to detect wheel set-bearing faults; however, its processing accuracy could degrade due to possible over-decomposition. A particle swarm method was suggested in [21] to optimize the VMD parameters, but the ratio errors with respect to the average value and the variance could decrease impact component accuracy. In addition, some other optimization algorithms were adopted in [22,23] to update the number of modes in VMD analysis for some specific applications. However, these mode selection algorithms still have some problems, such as slow convergence of parameter optimization due to complex spectrum distribution and trapping due to local minima.

To tackle the above limitations in VMD analysis, the second objective of this work is to propose a new VMD method, called modified VMD or MVMD, for nonlinear signal analysis and bearing fault detection. It is a great extension of the related work of the authors’ research team. For example, in comparison with the smart sensors in [4,5], the smart sensors developed in this work have a wider bandwidth and higher resolution. On the other hand, the adaptive VMD method in [6] applies the first IMF for bearing fault detection. Different from our previous related work, the novelties of the proposed MVMD technique include: (1) a new smart sensor-based DAQ system is developed for wireless vibration data collection, which has a wider measurement bandwidth and higher processing resolution accuracy; (2) in the proposed MVMD technique, a new correlation kurtosis approach is suggested to select the three most representative IMFs and formulate a new analytical signal. An envelope spectrum analysis is performed to recognize the representative features for bearing fault detection. The effectiveness of the developed smart sensor DAQ systems and the MVMD technique is verified by systematic experimental tests.

The remainder of the paper is organized as follows: The developed smart sensor DAQ system is described in Section 2. The proposed MVMD technique is discussed in Section 3. The effectiveness of the MVMD technique is examined in Section 4 by experimental tests. 

## 2. Development of Smart Sensor-Based DAQ System

**Figure 1:**
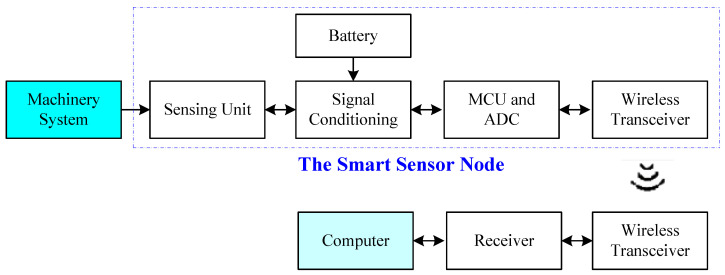
Block diagram of the smart sensor-based DAQ system.

A DAQ system is used to collect signals in different forms. A smart sensor node is a system that integrates the sensing units and signal conditioning functions (e.g., power supplies, amplification, adjustment, filtering, etc.) for data collection [3]. The related functions are controlled by a microcontroller unit (MCU) through a communications interface [4,5]. Wireless smart sensors are preferred in industrial applications to reduce costs and prevent DAQ cable distribution problems. The architecture of the developed smart sensor DAQ system is illustrated in Figure 1. The signals are measured using proper sensing units. After proper pre-conditioning operations, signals are digitized by the analog-to-digital conversion (ADC) unit integrated in the MCU. The digital vibration signals are transmitted to the receiver wirelessly, and then stored and processed in the computer for machine fault detection and diagnosis. 

### 2.1. Sensing Unit

The ICP (Integrated Circuit Piezoelectric) piezoelectric accelerometer is used as the sensing unit in this work (model HA1N100 from Hangjia Technology, Nanjing, China). Its frequency range is over [0.5, 10,000] Hz, with a sensitivity of 100 mV/g, and measurement range of ±50 g. This sensor requires an excitation DC voltage over [18 V, 28 V] and a constant current source over [2 mA, 20 mA].

### 2.2. Signal Conditioning Circuitry

**Figure 2:**
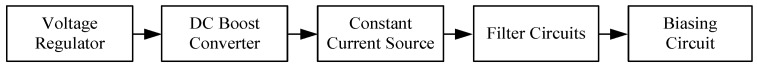
Block diagram of signal conditioning circuitry for a smart vibration sensor.

The signal conditioning circuitry is used to support the sensor requirements and process the analog data. The smart sensor node has a voltage regulated output, a boost converter, a constant current source, anti-aliasing filters, as well as other related circuits. Figure 2 shows the block diagram of the developed signal conditioning circuitry.

The developed smart sensor node is a battery-powered unit; its input voltage may vary with time, which can cause interruptions in operation. To prevent this problem, a constant voltage supply is needed. A voltage regulator (LD1117, from ST Microelectronics, Aliso Viejo, CA, USA) is selected to provide a constant 3.3 VDC voltage to the circuit [24]. The LD1117 is an adjustable voltage regulator, which can reduce the influence of the power supply fluctuations on the operation of the smart sensor nodes. 

A boost converter is used to generate the excitation voltage of 18–24 VDC for the piezoelectric accelerometer sensing unit. In this work, LT1930 (from Linear Technology, Milpitas, CA, USA) is used as the DC boost converter [25]. The LT1930 has a 1.2 MHz switching frequency, and it can convert the 3.3 VDC input to a 24 VDC to signal conditioning circuitry to maintain operation stability of the sensing unit. 

A constant current source is required to provide a constant excitation current for piezoelectric accelerometers. The LM234 chip (from Texas Instruments, Dallas, TX, USA) is selected in this work [26], which can provide a constant excitation current between 2 mA and 20 mA. It has good current regulation and wide dynamic voltage ranges from 1 V to 40 V, with an accuracy of ±3%. 

**Figure 3:**
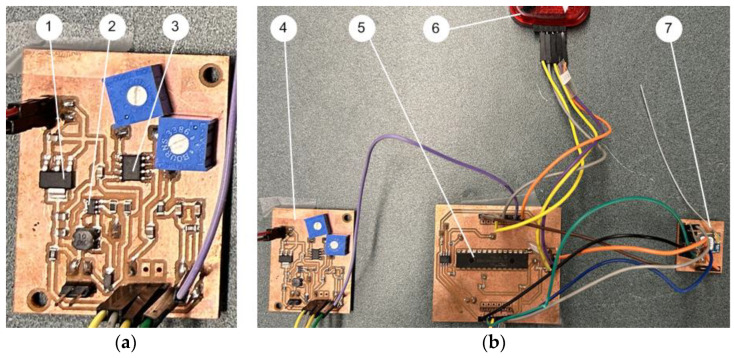
(**a**) Signal conditioning circuitry on a testing PCB board (two-layer): (1) Voltage regulator LD1117; (2) DC boost converter LT1930; (3) Constant current source LM234. (**b**) The smart sensor prototype: (4) The signal conditioning circuitry; (5) PIC32 MCU; (6) Pickit programmer; (7) CC1101 wireless transceiver.

Figure 3a shows the developed two-layer printed circuit board (PCB) testing board for the signal conditioning circuitry. 

### 2.3. Microcontroller PIC32MX

The MCU used in this smart sensor is PIC32MX250F128B (from Microchip Technology, Chandler, AZ, USA) [27]. PIC32 is a 32-bit MCU with 128 kB reprogrammable flash memory, 3 kB dedicated boot flash memory, and 32 kB SRAM. It can provide a 20 kHz sampling frequency, which can meet the bandwidth requirement in this work (i.e., 10,000 Hz). PIC32 also has a relatively low current consumption of 0.5 mA/MHz (versus 1.5 mA at 4 MHz for Atmega328p MCU). In addition, it can support UART, SPI, and I2C communications protocols. 

In general, machine condition monitoring is not conducted continuously, but periodically, for example, every 4 h. The MCU will provide sleep mode control to shut down the power system in the smart sensor nodes to save battery power when no data collection operation is conducted. 

### 2.4. SRAM 

An extra SRAM is needed in the developed wireless sensor DAQ system to store the digital data after the ADC and before wireless transmission. The 23LC1024 (from Microchip) SRAM chip is selected as the extra memory in this work. A single SRAM chip features 131,072 × 8-bit data points, and its memory for 10-bit ADC is 65,536 data points. If more data storage capacity is required, multiple SRAM chips can be used as slave devices. 

### 2.5. Wireless Communication

The wireless transmitter chip CC1101 transceiver (from Texas Instruments) [28] is used for wireless communication. CC1101 is a low-power and sub-1 GHz transmitter designed for different short-range frequency bands at 315 MHz, 433 MHz, 868 MHz, and 915 MHz. In this work, a 915 MHz ultra-high frequency band is chosen, which is assigned for industrial, scientific, and medical applications in North America. CC1101 can also provide extensive hardware support for functionalities.

The receiver can accept the digital data from different sensor nodes, and transmit the data to a computing unit, such as an analyzer or a computer. It can also control the operations of the smart sensor nodes for data acquisition processes, such as data collection intervals, sampling frequency, data transmission rate, sleep mode control, etc. The receiver unit uses the same type of MCU and wireless module as used in smart sensor nodes in this case.

**Figure 4:**
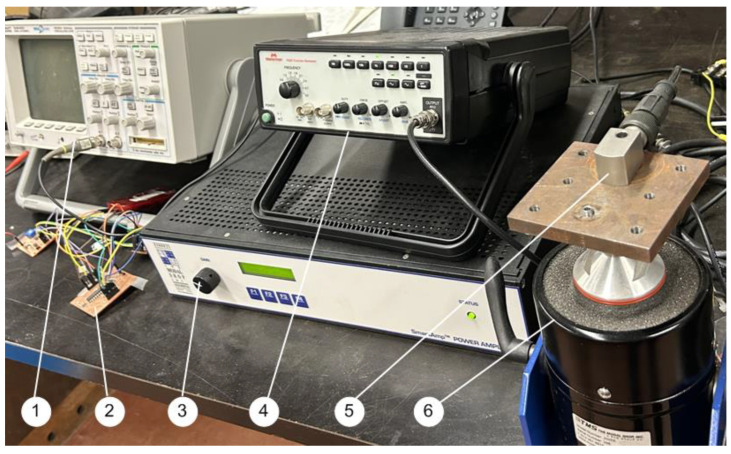
Experimental setup for testing the smart sensors: (1) Oscilloscope, (2) Tested two-layer PCB board, (3) Power amplifier, (4) Frequency generator, (5) Vibration sensing unit, (6) Excitation shaker.

The maximum transmission distance between a smart sensor node and the receiver depends on factors such as obstacles and noise interference. The maximum distance for using the C1101 ZigBee wireless communication protocol with 915 MHz is up to 500 m in an open space. Figure 4 shows the smart sensor node (a primary prototype for testing).

### 2.6. Performance Evaluation of the Smart Sensors

**Figure 5:**
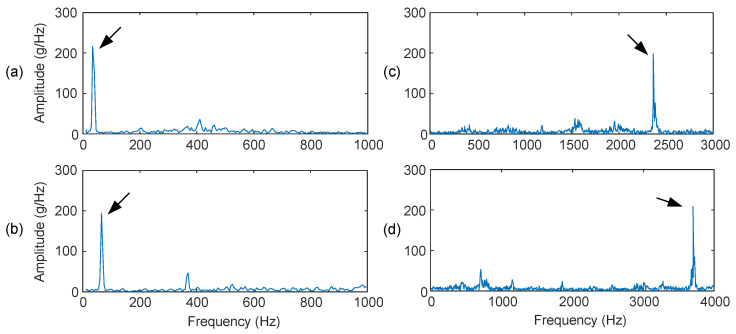
Amplitude spectra of collected signals using the smart vibration sensor prototype at different supply frequencies: (**a**) 30 Hz, (**b**) 60 Hz, (**c**) 2.3 K Hz, and (**d**) 3.7 K Hz. The arrows specify the excitation frequencies.

To evaluate the accuracy of the developed smart vibration sensor nodes, systematic tests are conducted using the experimental setup, as shown in Figure 4. The signal generator provides an excitation signal with a specified frequency. After amplification, the signal is fed to the shaker to generate the vibration signal with the specified frequency and mode used in the testing. Figure 5 shows some spectrum of the collected vibration signals at 30 Hz, 60 Hz, 2.3 kHz, and 3.7 kHz, respectively. The extra spectral components are due to noise, for example, generated by the parts on the table and shaker connecting frequencies. It is seen that input signals can be acquired accurately by the smart sensor node. 

**Figure 6:**
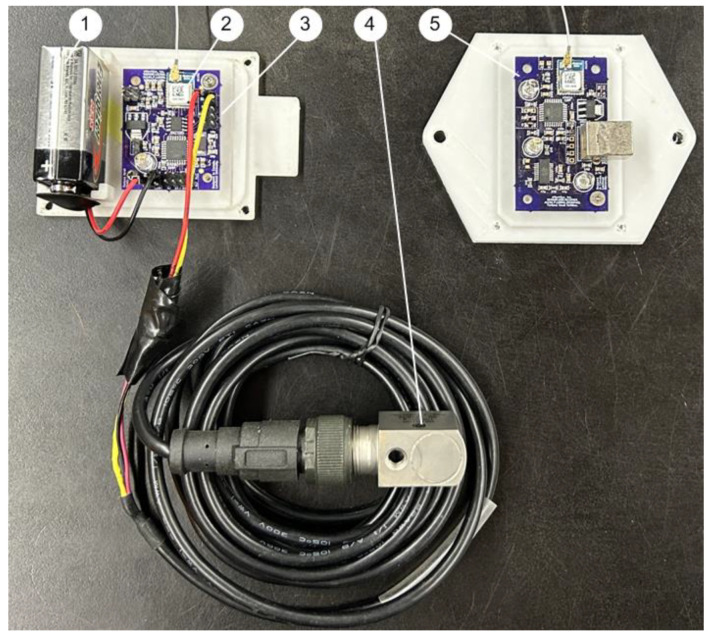
Developed wireless smart vibration sensor node (four-layer): (1) Battery, (2) Wireless transmitter, (3) A smart sensor node, (4) Vibration sensing unit, (5) Receiver.

Figure 6 shows the final prototype of the developed smart sensor DAQ system with four-layer PCBs, which has been used successfully in industrial applications.

## 3. MVMD Technique for Bearing Fault Detection

A new signal processing technique, modified VMD or MVMD, is proposed in this work for bearing fault detection. The MVMD technique consists of the following processing steps: (1) VMD analysis to generate intrinsic mode function (IMFs), (2) selecting most representative IMFs and reconstruct the signal, and (3) envelope spectral analysis for bearing fault detection. Details will be discussed in this section. 

### 3.1. VMD Processing

VMD is an adaptive time–frequency analysis method that can process nonlinear and nonstationary signals [16]. In processing, the VMD decomposes a signal x(t) into a series of IMFs, each having a finite bandwidth and located around a center frequency. An IMF, $u_{k}(t)$, can be expressed as:

(1) $$u_{k}(t)=A_{k}(t)\cos(\varphi_{k}(t)),\;k\;=\;1,\;2,\;\ldots ,\;K$$

where $A_{k}(t)$ is the instantaneous amplitude; $\varphi_{k}(t)$ in rad is the phase angle; *K* is the number of IMFs considered. The instantaneous frequency $\omega_{k}(t)=\frac{d\varphi_{k}(t)}{dt}$ in rad/sec.

The VMD aims to solve a constrained variational problem represented by:

(2) $$\min_{\left\{u_{k}\},\{\omega_{k}\right\}}\left\{\sum_{k=1}^{K}{\left\|\partial_{t}\left[\left(\delta (t)+\frac{j}{\pi t}\right)*u_{k}(t)\right]e^{-j\omega_{k}t}\right\|}_{2}^{2}\right\}$$

where $\sum_{k=1}^{K}u_{k}(t)=x(t)$; $\partial_{t}$ represents the partial derivative of time; $\delta =2\pi /\omega_{k}(t)$ is the Dirac delta distribution; *j* is the complex number; * is the convolution operator.

Equation (2) can be converted into an unconstrained optimization problem using the augmented Lagrangian method [6,16]. An optimal solution can be obtained using an alternated direction method of multipliers [16]. Thus, mode $u_{k}$ can be updated by:

(3) $$u_{k}^{n+1}=\arg_{u_{k}\in F}\min\{\alpha_{k}{\left\|\partial_{t}\left[\left(\delta (t)+\frac{j}{\pi t}\right)*u_{k}(t)\right]e^{-j\omega_{k}t}\right\|}_{2}^{2}+{\left\|x(t)-\sum_{i}u_{i}(t)+\frac{\lambda (t)}{2}\right\|}_{2}^{2}\}$$

where $\alpha_{k}$ is a penalty factor and λ(t) is Lagrangian multiplier. Using Parseval/Plancherel Fourier isometry under the *L*^2^ norm and the Hermitian symmetry of the FT, Equation (3) can be solved in the frequency domain:

(4) $$U_{k}^{n+1}=\arg_{U_{k},u_{k}\in F}\min\{\int_{0}^{\infty }4\alpha_{k}{\left(\omega -\omega_{k}\right)}^{2}{\left|U_{k}(\omega )\right|}^{2}+2{\left|X(\omega )-\sum_{i}U_{i}(\omega )+\frac{\Lambda (\omega )}{2}\right|}^{2}d\omega \}$$

where $u_{k}(t)↔U_{k}(\omega )$, x(t)↔X(ω), λ(t)↔Λ(ω) and the related FT pairs. 

This quadratic optimization problem can also be represented as [29]:

(5) $$U_{k}^{n+1}(\omega )=\frac{X(\omega )-\sum_{i\neq k}U_{i}(\omega )+\frac{\Lambda (\omega )}{2}}{1+2\alpha_{k}{\left(\omega -\omega_{k}\right)}^{2}}$$

In addition, the center frequency $\omega_{k}$ of IMF mode $u_{k}$ can be updated by:

(6) $$\omega_{k}^{n+1}=\arg_{\omega_{k}}\min\left\{{\left\|\partial_{t}\left[\left(\delta (t)+\frac{j}{\pi t}\right)*u_{k}(t)\right]e^{-j\omega_{k}t}\right\|}_{2}^{2}\right\}$$

Similarly, the center frequency can also be optimized in the frequency domain by:

(7) $$\omega_{k}^{n+1}=\arg_{\omega_{k}}\min\left\{\int_{0}^{\infty }{\left(\omega -\omega_{k}\right)}^{2}{\left|U_{k}(\omega )\right|}^{2}d\omega \right\}$$

This quadratic optimization problem can be solved by:

(8) $$\omega_{k}^{n+1}=\frac{\int_{0}^{\infty }\omega {\left|U_{k}(\omega )\right|}^{2}d\omega }{\int_{0}^{\infty }{\left|U_{k}(\omega )\right|}^{2}d\omega }$$

The Lagrangian multiplier can be updated by:

(9) $$\Lambda^{n+1}=\Lambda^{n}\left(\omega \right)+\tau \left(X(\omega )-\sum_{k}U_{k}^{n+1}(\omega )\right)$$

where *τ* is the iteration step size (*τ* = 0 is selected in this case). The iteration will be terminated if the errors meet the following convergence condition:

(10) $$\sum_{k}\frac{{\left\|U_{k}^{n+1}-U_{k}^{n}\right\|}_{2}^{2}}{{\left\|U_{k}^{n}\right\|}_{2}^{2}}<\varepsilon$$

where *ε* is the convergence threshold (*ε* = 0.01 is selected in this case). 

### 3.2. Determination of the Penalty Factor

The penalty factor $\alpha_{k}$ is used to determine the bandwidth of the mode component [16]. In general, a higher penalty factor will generate a narrower bandwidth of the mode component, and vice versa. According to the spectral characteristics of different bearing fault representative features, the penalty factor $\alpha_{k}$ will be empirically estimated by [6]:

(11) $$\alpha_{k}=\left(\frac{1}{1+e^{\log_{10}\frac{2f_{kc}}{f_{s}}}}-0.5\right)\cdot \frac{f_{s}}{2}$$

where *f_kc_* is the center frequency of the *k*-th mode component; *f_s_* is the sampling frequency. 

Based on Equation (8), the discrete form of frequency *f_kc_* in Hz can be calculated by:

(12) $$f_{kc}=\frac{\sum_{n=1}^{N/2}\left[n\cdot f_{s}/N\cdot {\left|U_{k}[n]\right|}^{2}\right]}{\sum_{n=1}^{N/2}{\left|U_{k}[n]\right|}^{2}}$$

where *N* is the length of the vibration signal, and $U_{k}[n]$ is the discrete FT of the *k*-th mode component *u_k._*

### 3.3. A Correlation Kurtosis Method for IMF Section and Signal Reconstruction

VMD analysis will be used to select the IMFs containing the fault characteristic features. Some IMFs are more sensitive to machine faults than others. A correlation kurtosis method is proposed to screen the most sensitive IMFs related to machine faults so as to improve the accuracy of fault diagnosis, while improving processing efficiency by using fewer representative IMFs.

Rolling element bearings have periodic impact components in the vibration signal when a bearing component is damaged. The kurtosis is sensitive to tail modulation in probability distribution functions but less sensitive to machine operating conditions in terms of speed and load. When the kurtosis value of an IMF is large, it usually indicates that this IMF would contain more characteristic features related to bearing defects. The kurtosis $K_{U,k}$ of the IMF $u_{k}$ in the discrete form, $u_{k}[n]$, can be calculated by:

(13) $$K_{U,k}=\frac{\frac{1}{N}\sum_{n=1}^{N}{\left(u_{k}[n]-{\bar{u}}_{k}\right)}^{4}}{\sigma_{k}^{4}}$$

where $\sigma_{k}$ and ${\bar{u}}_{k}$ are the standard deviation and mean of $u_{k}[n]$, *n* = 1, 2, …, *N* and *N* is the length of the IMF signature.

In general, the more IMFs that are used for analysis, the higher the processing accuracy, but it will take a longer time for processing. To improve processing efficiency, based on a systematic investigation, the first ten IMFs will be selected in this work for analysis (i.e., *K* = 10), without using the general recursive selection algorithm for *K* selection [16] so as to further reduce processing time. However, when a bearing is damaged, the representative features may time-vary, especially considering slip among bearing components [5,6]. In this work, a new correlation kurtosis method will be applied to rank IMF $u_{k}[n]$ as described below.

Cross-correlation is a measure of similarity between two signals even if they have phase differences from one another. In this work, a normalized cross-correlation is used for analysis. The correlation indicator $C_{k}$ is computed by

(14) $$C_{k}=\frac{1}{N}\sum_{n=1}^{N}\frac{E\left[\left(x-\mu_{x}\right)\left(u_{k}-\mu_{u_{k}}\right)\right]}{\sigma_{x}\sigma_{u_{k}}}$$

where *x* is the input vibration signal; $u_{k}$ is the IMF; $\mu_{x}$ and $\mu_{u_{k}}$ represent the mean values of *x* and $u_{k}$, respectively; $\sigma_{x}$ and $\sigma_{u_{k}}$ are the corresponding standard deviation values; *E*[.] denotes the expectation operator. 

In the proposed MVMD technique, IMFs are ranked based on the weight factor, $W_{k}$, associated with both IMF kurtosis and its correlation with the original signal:

(15) $$W_{k}=C_{k}K_{U,k}$$

If three IMFs with the highest weight factor values are selected to reconstruct the signal such that:

(16) $$x^{\prime }=\sum_{m=1}^{3}{\bar{W}}_{m}u_{m}$$

where ${\bar{W}}_{m}=\frac{W_{m}}{\sum_{m=1}^{3}W_{m}}$. The analytical signal can be formulated by using the Hilbert transform:

(17) $$x_{a}=x^{\prime }+jH(x^{\prime })=A_{x}e^{j\varphi_{x}}$$

where *H*(.) represents the Hilbert transform; $\varphi_{x}=\arctan\left(H(x^{\prime })/x^{\prime }\right)$ is the instantaneous phase; $A_{x}=\sqrt{{\left(x^{\prime }\right)}^{2}+{\left(H(x^{\prime })\right)}^{2}}$ is the instantaneous amplitude (i.e., signal envelope).

**Figure 7:**
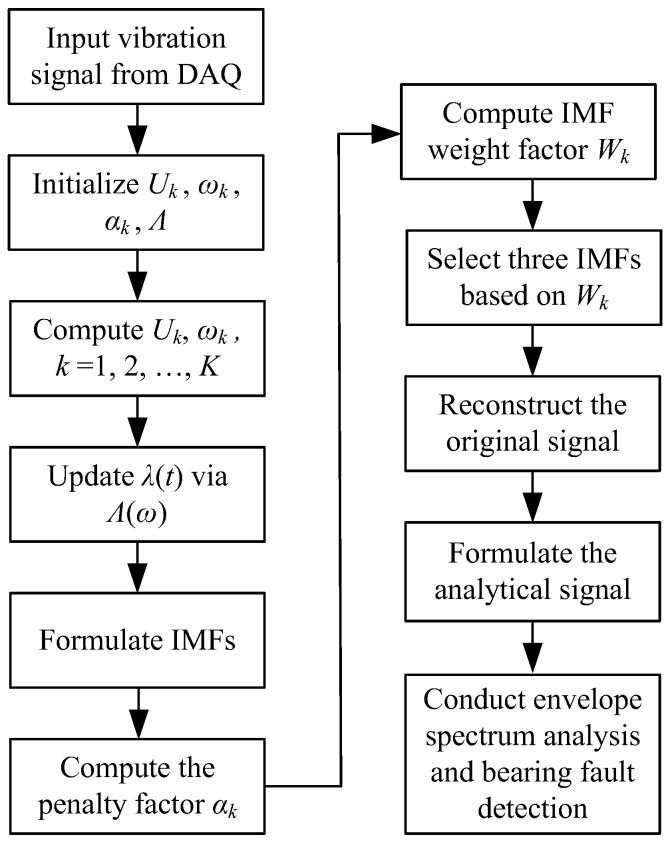
Flowchart of the processing procedures of the MVMD technique.

The envelope spectrum is obtained by taking the discrete FT of the envelope signal. Bearing fault detection is undertaken by examining characteristic frequency information on the envelope spectrum. Figure 7 shows the flowchart of the processing of the MVMD technique, and the processing procedures are summarized as follows:

Step 1: Initialize {$U_{k}^{1}$}, {$\omega_{k}^{1}$}, $\Lambda^{1}$, and *n* := 1.

Step 2: Calculate $U_{k}$ and $\omega_{k}$ using Equations (5) and (8), respectively, *k* = 1, 2, …, *K*, (*K* = 10). 

Step 3: Update Λ in Equation (9).

Step 4: Repeat Steps 2–3 until the iteration meets the criterion in Equation (10).

Step 5: Compute IMFs $u_{k}$, *k* = 1, 2, …, *K.*

Step 6: Compute the penalty factor using Equation (11)

Step 7. Compute the weight factor *W_k_* through Equation (15). 

Step 8: Reconstruct the signal using Equation (16).

Step 9: Formulate the analytical signal using Equation (17).

Step 10: Conduct envelope spectrum analysis and perform bearing fault detection.

## 4. Experimental Tests and Data Analysis

The effectiveness of the proposed MVMD technique will be evaluated experimentally in this section. 

### 4.1. Experimental Setup

**Figure 8:**
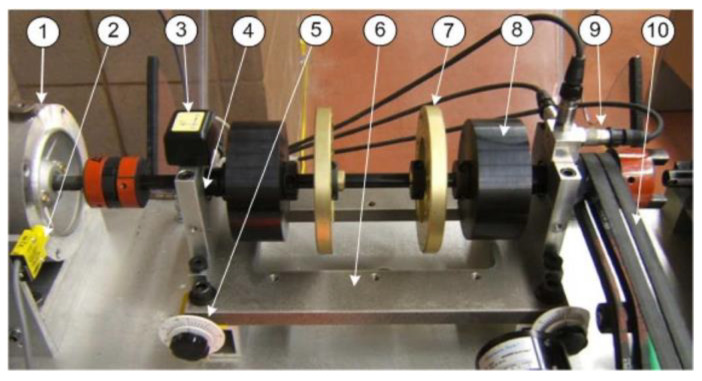
Experimental apparatus used in this work: (1) The drive motor; (2) An optical transducer (3) Smart vibration sensor; (4) Bearing housing; (5) Shaft misalignment controller; (6) Shaft system platform; (7) Small radial load disc; (8) Heavy radial load disc; (9) ICP accelerometers; (10) Dynamic load drive unit.

The experimental setup used in this paper is shown in Figure 8. The system is driven by a 3 HP motor, and its speed ranges from 100 to 3600 r/min. The shaft rotation is controlled by using a speed controller. An optical transducer is used to provide a one-pulse-per-revolution signal to measure the shaft speed. Two ball rolling element bearings are fitted in the bearing housings for testing. The developed smart sensor node is mounted on one of the bearing housings to measure vibration signals. The collected signals are transmitted wirelessly to the receiver that is connected to a computer via a USB port. To make a comparison, vibration signals are also collected using three commercially available ICP accelerometers mounted along three directions on another bearing housing. The signals collected by these ICP accelerometers and the optical transducer are fed to the computer through a data acquisition board (NI PCI-4472) for further signal processing. The static loading to the bearings is applied by load disks. The dynamic load is applied by a magnetic brake system through a belt drive. 

### 4.2. Performance Evaluation

Deep-groove ball bearings (MB ER-10 K) are tested with four bearing conditions: healthy/normal bearings, bearings with outer race defect, inner race defect, and rolling element fault. The tested bearings have the following parameters:

- the number of rolling elements: 8;
- rolling element diameter: 7.938 mm;
- pitch diameter: 33.503 mm;
- the angle of contact: 0 degree.

The tests are undertaken with a shaft speed from 600 rpm to 3600 rpm, with the load at light, medium, and heavy levels. Some typical processing results corresponding to shaft rotating frequency *f_r_* ≈ 30 Hz (i.e., approximately 1800 rpm) at medium load level (i.e., 6.5 Nm) are used for illustration. The sampling frequency is 32,000 Hz. The theoretical characteristic frequencies corresponding to different bearing health conditions are calculated using the related dynamics equations [1] and are summarized in Table 1. For comparison, the test results of the proposed MVMD technique will be compared with the related techniques, that is, the HHT [8] and a self-adaptive VMD as proposed in [23]. All the techniques are implemented in MATLAB.

To quantitatively compare the performance of the related fault detection techniques, a fault indicator *F_I_* is introduced for characterize feature properties:

(18) $$F_{I}=\sum_{h}\frac{A_{h}-\mu_{A}}{\mu_{A}}$$

where $A_{h}$ is the spectral component amplitude of the *h*-th harmonic of the characteristic frequency that can be recognized; $\mu_{A}$ is the average spectral value over the bandwidth of interest (400 Hz in this case).

(1) Processing results for a healthy bearing

**Figure 9:**
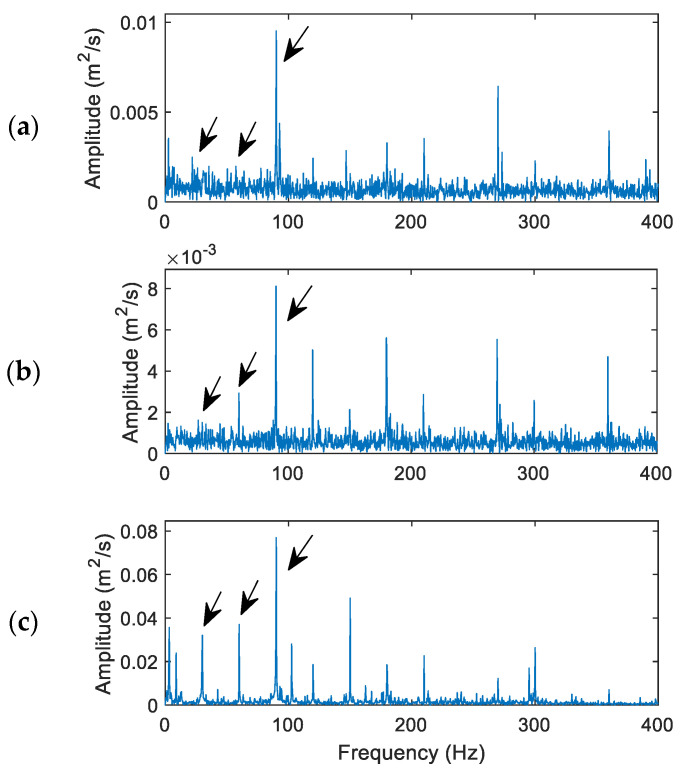
Performance comparison of the related techniques for a healthy bearing: (**a**) the HHT, (**b**) the VMD, (**c**) the proposed MVMD. Arrows indicate characteristic frequency (30 Hz) and its harmonics. The arrows indicate bearing characteristic frequency (30 Hz) and its harmonics.

Figure 9 shows processing result comparison for a healthy bearing using the related techniques. Its characteristic frequency *f_H_* = 30 Hz in this case and the updated MVMD penalty factor is 932, calculated using Equation (11). The selected IMFs are IMF_1_, IMF_2_, and IMF_4_.

It is seen that the dominant spectral component is the third harmonic of the characteristic frequency (approximately 90 Hz). All these three techniques can recognize this harmonic clearly. However, the proposed MVMD in Figure 9c can also recognize the fundamental frequency (30 Hz) and its second harmonic (60 Hz), due to its effective IMF processing and denoising operations. The VMD in Figure 9b performed better than the HHT in Figure 9a with the recognized second harmonic (60 Hz), due to its more efficient IMF selection and processing than the HHT. Based on Equation (18), the fault indicator values are 3.75, 4.22, and 5.09 for the HHT, VMD, and MVMD techniques, respectively.

(2) Processing results for a bearing with outer race defect

**Figure 10:**
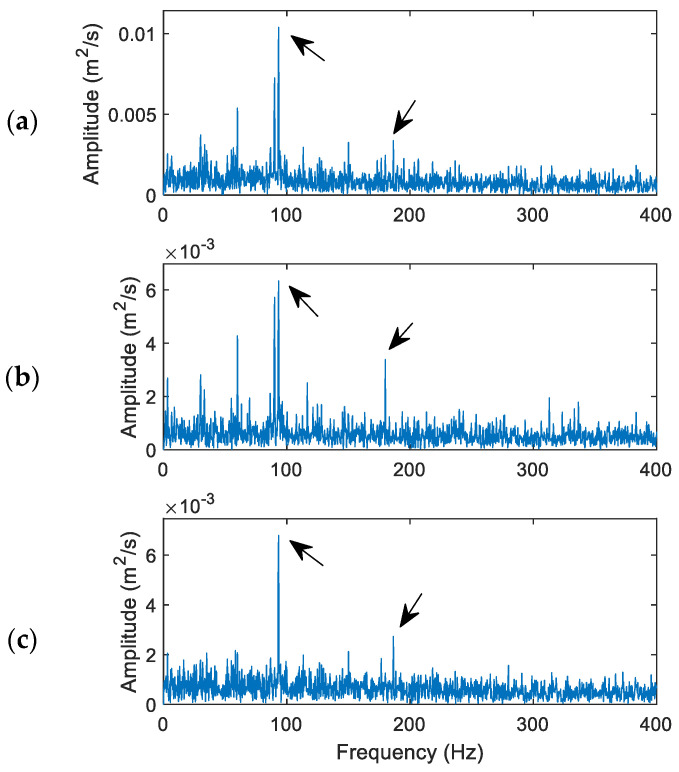
Performance comparison of the related techniques for a bearing with outer race defect: (**a**) the HHT, (**b**) the VMD, (**c**) the proposed MVMD. Arrows indicate characteristic frequency (90.9 Hz) and its harmonics.

Figure 10 shows the processing results and performance comparison using the related techniques for a bearing with outer race damage. The characteristic frequency is *f_od_* = 90.9 Hz, and the updated penalty factor is 971 in the MVMD technique. The selected IMFs are IMF_1_, IMF_2_, and IMF_3_.

In this case, all three techniques can recognize the characteristic frequency (90.9 Hz) for the bearing outer race fault. This is because when the bearing outer race (fixed ring) is damaged, the generated impulses and features are usually time-invariant, which are relatively easy to extract using general fault detection techniques. The VMD in Figure 10b performs better than the HHT in Figure 10a, with a clear second harmonic due to its more efficient IMF processing in signal demodulation. However, the fundamental characteristic frequency components (90.9 Hz) using both the HHT and the VMD contain adjacent spectral components due to modulation of other vibration signals, which may generate false diagnosis especially in automatic bearing health monitoring. On the other hand, the proposed MVMD technique provides the best diagnostic result in this case, as demonstrated in Figure 10c, due to its more effective IMF processing and denoising operation. In this case, the fault indicator values calculated by Equation (18) are 2.94, 3.30, and 5.71 for the HHT, VMD, and MVMD techniques, respectively.

(3) Processing results for a bearing with inner race defect

**Figure 11:**
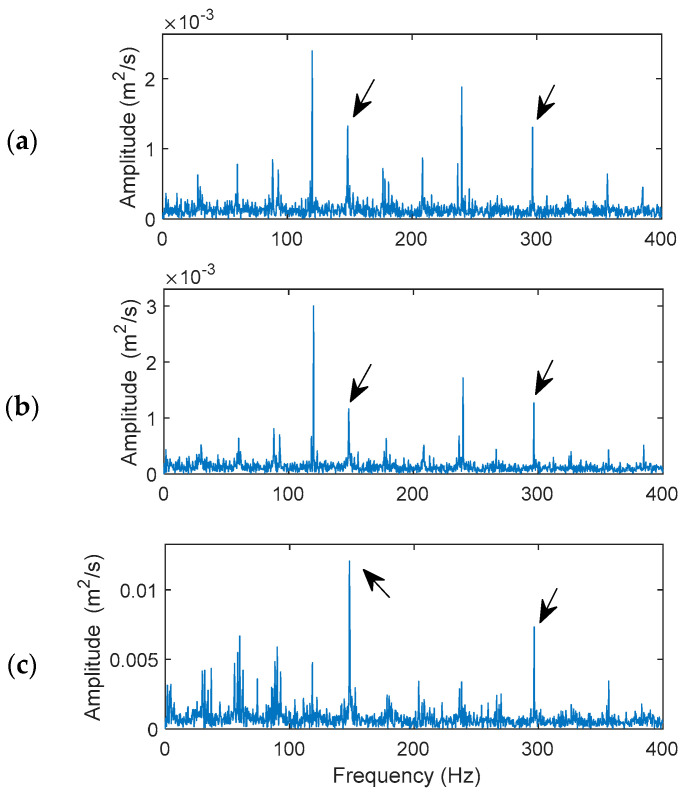
Performance comparison of the related techniques for a bearing with inner race fault: (**a**) the HHT, (**b**) the VMD, (**c**) the proposed MVMD. Arrows indicate characteristic frequency (147.9 Hz) and its harmonics.

Figure 11 shows the processing results and performance comparison using the related techniques for a bearing with an inner defect. The characteristic frequency is *f_id_* = 147.9 Hz. The updated penalty factor in the MVMD is 1210, and the selected IMFs are IMF_1_, IMF_3_, and IMF_4_.

As stated before, bearing fault detection on a rotating inner race and a rolling element is more challenging because the defect-related impulse resonance features vary with time, especially considering the slip effects among bearing components. Although the HHT in Figure 11a and VMD techniques in Figure 11b can recognize the occurrence of a bearing inner race defect, their characteristic frequency (147.9 Hz) and/or the second harmonics do not dominate the spectral maps. However, the proposed MVMD technique provides the best performance in this case, which can clearly predict the bearing fault condition, with the domain fault characteristic frequency (147.9 Hz) and its second harmonic as illustrated in Figure 11c. Its higher resolution and better fault diagnostic accuracy are due to its feature enhancement effects and efficient IMF feature demodulation. In this case, the fault indicator values are 0.72, 0.65, and 3.87 for the HHT, VMD and MVMD techniques, respectively.

(4) Processing results for a bearing with rolling element defect

**Figure 12:**
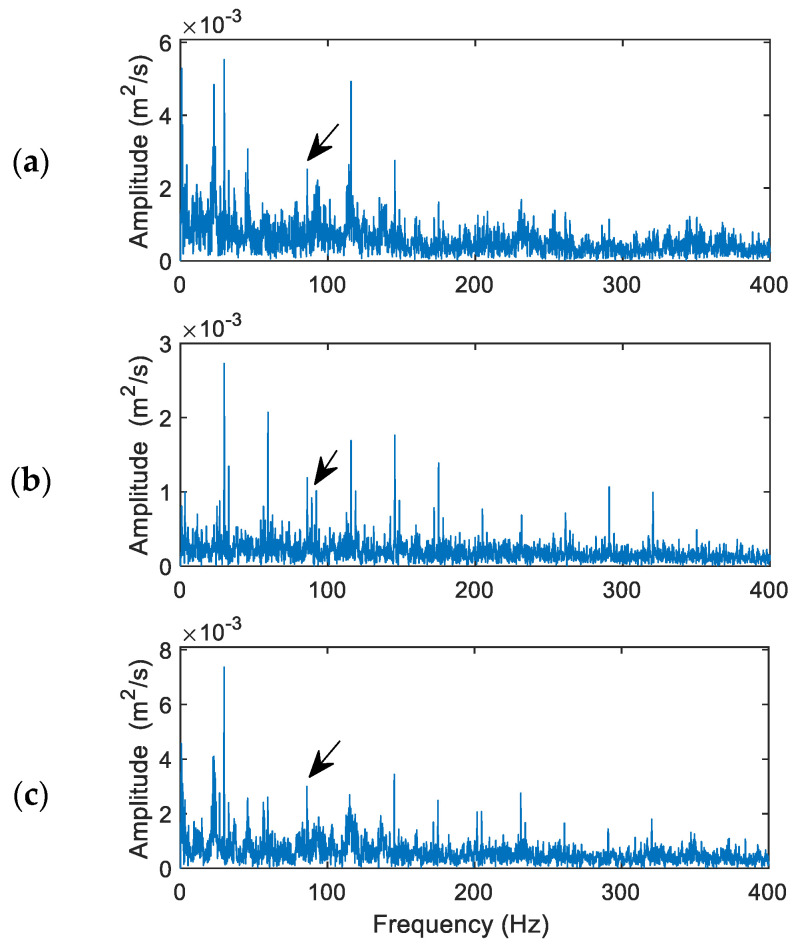
Performance comparison of the related techniques for a bearing with rolling element fault: (**a**) the HHT, (**b**) the VMD, (**c**) the proposed MVMD. Arrows indicate characteristic frequency (91.57 Hz) and its harmonics.

Figure 12 shows the processing results and performance comparison using the related techniques for a bearing with rolling element damage. The theoretical characteristic frequency is *f_bd_* = 91.57 Hz. The MVMD has an updated penalty factor 288, and the selected IMFs are IMF_1_, IMF_2_, and IMF_5_.

In general, bearing fault detection in a rolling element could be the most challenging task because the characteristic features are time-varying. In this case, both the HHT in Figure 12a and VMD in Figure 12b have failed to identify the characteristic fault frequency (91.57 Hz) clearly, even though the HHT performs a little better than the VMD in this case. They cannot effectively demodulate the representative features from the collected vibration signal. The MVMD, on the other hand, is the only technique that can recognize the fault characteristic frequency (91.57 Hz) in this case, as shown in Figure 12c, even though it is not the dominant frequency component on the spectral map. In this case, the fault indicator values are 0.39, 0.14, and 0.57 for the HHT, VMD, and MVMD techniques, respectively.

In addition, the processing results using the vibration signals collected by the smart sensor DAQ system are almost identical with those vibration signals collected by using the commercial ICP accelerometers (along the vertical direction in this case). It demonstrates that the developed smart sensors and DAQ system can be applied reliably for data collection. 

## 5. Conclusions

A new smart sensor-based monitoring technology has been developed in this work for real-time machine condition monitoring and fault detection. As rolling element bearings are commonly used in rotating machines, and most machine imperfections are related to bearing defects, this work focuses on bearing fault detection. Firstly, a smart sensor-based DAQ system is developed for wireless vibration signal collection. Secondly, a MVMD technique is proposed for nonstationary signal analysis and bearing fault detection. The proposed MVMD technique takes several processing steps: (1) the signal is decomposed into a series of IMFs; (2) a correlation kurtosis method is suggested to choose the most representative IMFs and construct the analytical signal; (3) envelope spectrum analysis is conducted to identify the representative features for bearing fault detection. The effectiveness of the developed smart sensor DAQ system and the MVMD technique is examined by systematic experimental tests. Test results show that the developed smart sensor DAQ system can collect vibration signals accurately. The proposed MVMD technique outperforms the related techniques under tested bearing conditions. This monitoring technology has the potential to be applied to machine condition monitoring and bearing fault detection in industrial applications.

## Figures and Tables

**Table 1:** The bearing fault characteristic frequencies in terms of shaft speed *f_r_*.

	Characteristic Frequency (Hz)
Healthy bearing	*f_H_* = *f_r_*
Outer race fault	*f_od_* = 3.052 × *f_r_*
Inner race fault	*f_id_* = 4.947 × *f_r_*
Rolling element fault	*f_bd_* = 3.983 × *f_r_*

## Data Availability

Data are contained within the article.
